# Modelling the endothelial blood-CNS barriers: a method for the production of robust *in vitro* models of the rat blood-brain barrier and blood-spinal cord barrier

**DOI:** 10.1186/1471-2202-14-59

**Published:** 2013-06-18

**Authors:** P Marc D Watson, Judy C Paterson, George Thom, Ulrika Ginman, Stefan Lundquist, Carl I Webster

**Affiliations:** 1 MedImmune Ltd, Granta Park, Cambridgeshire CB21 6HG, UK; 2 AstraZeneca, Södertälje SE 15185, Sweden

**Keywords:** Blood-brain barrier, Blood-spinal cord barrier, *in vitro*, TEER, Drug discovery, Permeability coefficient, FITC-dextran, Lucifer yellow, Hydrodynamic radius

## Abstract

**Background:**

Modelling the blood-CNS barriers of the brain and spinal cord *in vitro* continues to provide a considerable challenge for research studying the passage of large and small molecules in and out of the central nervous system, both within the context of basic biology and for pharmaceutical drug discovery. Although there has been considerable success over the previous two decades in establishing useful *in vitro* primary endothelial cell cultures from the blood-CNS barriers, no model fully mimics the high electrical resistance, low paracellular permeability and selective influx/efflux characteristics of the *in vivo* situation. Furthermore, such primary-derived cultures are typically labour-intensive and generate low yields of cells, limiting scope for experimental work. We thus aimed to establish protocols for the high yield isolation and culture of endothelial cells from both rat brain and spinal cord. Our aim was to optimise *in vitro* conditions for inducing phenotypic characteristics in these cells that were reminiscent of the *in vivo* situation, such that they developed into tight endothelial barriers suitable for performing investigative biology and permeability studies.

**Methods:**

Brain and spinal cord tissue was taken from the same rats and used to specifically isolate endothelial cells to reconstitute as *in vitro* blood-CNS barrier models. Isolated endothelial cells were cultured to expand the cellular yield and then passaged onto cell culture inserts for further investigation. Cell culture conditions were optimised using commercially available reagents and the resulting barrier-forming endothelial monolayers were characterised by functional permeability experiments and *in vitro* phenotyping by immunocytochemistry and western blotting.

**Results:**

Using a combination of modified handling techniques and cell culture conditions, we have established and optimised a protocol for the *in vitro* culture of brain and, for the first time in rat, spinal cord endothelial cells. High yields of both CNS endothelial cell types can be obtained, and these can be passaged onto large numbers of cell culture inserts for *in vitro* permeability studies. The passaged brain and spinal cord endothelial cells are pure and express endothelial markers, tight junction proteins and intracellular transport machinery. Further, both models exhibit tight, functional barrier characteristics that are discriminating against large and small molecules in permeability assays and show functional expression of the pharmaceutically important P-gp efflux transporter.

**Conclusions:**

Our techniques allow the provision of high yields of robust sister cultures of endothelial cells that accurately model the blood-CNS barriers *in vitro*. These models are ideally suited for use in studying the biology of the blood-brain barrier and blood-spinal cord barrier *in vitro* and for pre-clinical drug discovery.

## Background

The endothelial blood-CNS barriers, located at the microvascular cells of the brain and spinal cord, represent the crucial interface between the maelstrom of the peripheral circulation and the tightly regulated environment of the central nervous system (CNS). Here, the blood–brain barrier (BBB) and blood-spinal cord barrier (BSCB) present a formidable structural and metabolic barrier that partitions the CNS parenchyma. Far from being impenetrable blockades, the blood-CNS barriers are highly dynamic regulatory interfaces that apply strict control over the passage of blood-borne substances into the CNS, and oversee regulated transport of large and small molecules back into the periphery. The blood-CNS barriers are of great relevance to pharmaceutical drug discovery, as the BBB and BSCB present obstacles to the delivery of compounds aimed at the treatment of CNS disorders affecting the brain and spinal cord. A fuller understanding of each of these barriers will aid the development of CNS-targeted small and large molecule therapies to treat wide-ranging and devastating neurological diseases, from neurodegeneration to chronic pain [1-4]. To facilitate basic research and drug discovery, it is therefore highly desirable to have robust and convenient *in vitro* models of the BBB and BSCB, from species relevant for pre-clinical investigations [1,5]. Such models must aim to faithfully recreate the exquisite *in vivo* tissue microenvironment that induces a blood-barrier phenotype. For the BBB, as well as the more poorly understood BSCB, this has posed a considerable technical challenge. The goal for *in vitro* BBB and BSCB model development is to obtain convenient primary cell cultures that can be easily and inexpensively established and possess robust barrier phenotypes similar to those seen *in vivo*. Good *in vitro* barriers will possess properties such as high transendothelial electrical resistance (TEER) across the endothelial monolayer and low passive, non-specific paracellular permeability to small and large molecules such as Lucifer yellow (LY), hydrophobic compounds and FITC-labelled dextrans. For a truly representative model, other features such as expression of receptors and transporters on the endothelial cell surface and intracellular transcytosis machinery must be maintained to allow transcellular transport pathways for ions, small molecules, peptides and proteins to be reconstituted *in vitro*. An additional problem for establishing robust *in vitro* blood-CNS barrier models is the provision of sufficient numbers of cells to allow for rigorous characterisation of the models and investigative biology or drug screening. The typically low yields of endothelial cells can severely limit research efforts, particularly for tissues such as the spinal cord where the amount of tissue recovered per animal is especially low.

The fundamental features of the blood-CNS barriers *in vivo* are well known but difficult to fully replicate *in vitro*. These barrier-forming elements include highly developed endothelial tight junctions that lead to high TEER, lack of endothelial fenestrae, low non-specific pinocytosis and the expression of receptors and transporters that facilitate small and large molecule influx and efflux [6]. One of the greatest hurdles to translating these *in vivo* features into robust *in vitro* models is that the development of the *in vivo* CNS-blood barrier phenotype is exquisitely regulated by the cellular microenvironment of the brain and spinal cord endothelial cells. Astrocytes have long been demonstrated to induce barrier function at the BBB *in vitro* and *in vivo*[7] and increasing evidence is pointing to a similarly important role for pericytes in barrier development and maintenance [8-12]. In spite of these challenges, *in vitro* modelling of the BBB, and to a lesser extent the BSCB, has progressed significantly over the previous two decades. BBB primary endothelial cell culture models have been established with cells isolated from human [13-19], mouse [20-26], rat [16,27-35], bovine [36-43] and pig [44-54] brain tissues. BSCB endothelial models have, in contrast, currently only been described *in vitro* for a single species, namely mouse [55]. BBB *in vitro* primary cell culture barrier models have progressed from simple solo-cultures of brain endothelial cells to more complex co-culture models in which endothelial cells are grown on porous cell culture inserts and co-cultured with postnatal rodent astrocytes [7]. Astrocytes may be plated either into the bottom of a multi-well dish into which the insert is placed or grown on the underside of the insert itself in so-called back-to-back contact co-culture models. Recently, increasingly complex co-culture models, such as triple cultures of endothelial cells with astrocytes and pericytes [10-12] have been developed. However, although these models display good barrier phenotypes *in vitro*, they are particularly labour-intensive and expensive to establish. It has also been demonstrated that neural stem cells have the ability to induce barrier properties *in vitro* in a manner which may be representative of BBB development *in vivo*[56,57]. Further improvements to barrier phenotype have been demonstrated through the manipulation of cell culture conditions. It has been known for several years that factors such as modulators of intracellular cAMP signalling [58,59], glucocortocoids [22,26,53,60,61] and growth factors such as bFGF [62,63] can induce improvements in barrier phenotype in cultured primary brain endothelial cells. Other manipulations, such as modulating the buffering capacity of cell culture medium [64] and optimising endothelial cell seeding density [23,31] can influence and improve barrier function. In recent years, the inclusion of puromycin as a method for removing contaminating non-endothelial cells has become established as a key element of *in vitro* BBB cell culture protocols [27,31,51,61,65].

There continues to be a need, however, to evolve blood-CNS barrier modelling techniques to achieve increasingly representative *in vitro* phenotypes that faithfully recapitulate the tight, discriminative situation found in brain and spinal cord capillaries *in vivo*. The reproducibility of BBB cell culture models can be inconsistent from week-to-week or lab-to-lab, and thus for routine use in academic and pharmaceutical studies it is highly desirable to have protocols that produce robust and reliable *in vitro* barriers. Additionally, it is also highly useful to have such *in vitro* blood-CNS barrier models from commonly used pre-clinical species, such as the rat, so that *in vitro* data is relevant to the *in vivo* models employed during early CNS drug discovery efforts. Such representative *in vitro* models may then be employed to characterise drug toxicity and permeability early in pharmaceutical development and thus have great potential for contributing to a reduction in the high attrition rate of drugs in early development for CNS diseases.

We set out to investigate whether an easy and highly robust protocol could be established that allowed the production of large numbers of brain and, for the first time in rat, spinal cord endothelial cells from a minimal amount of starting tissue. The aim was to obtain high yields of cells that could be passaged onto cell culture inserts and induced to form tight monolayer barriers for permeability studies. By optimizing culture conditions using specific handling techniques and commercially available reagents, we have demonstrated the isolation and culture of large numbers of both types of endothelial cell, from the same animals. These barrier cultures are pure endothelial in nature, show correct localisation of tight junction proteins, have discriminating barrier characteristics and restrict the paracellular permeability of large and small molecules. We thus present a further evolution in the techniques for establishing *in vitro* blood-CNS barriers in a relevant pre-clinical species. These models have utility for investigation of the basic biology of the BBB and BSCB *in vitro* and in CNS-focused pharmaceutical drug development and toxicity studies.

## Methods

### Materials

All tissue culture media, supplements and reagents are from Gibco, Life Technologies UK, unless otherwise stated. All compounds and reagents are from Sigma-Aldrich, UK unless otherwise stated.

### Isolation of rat brain microvascular endothelial cells

**Figure 1:**
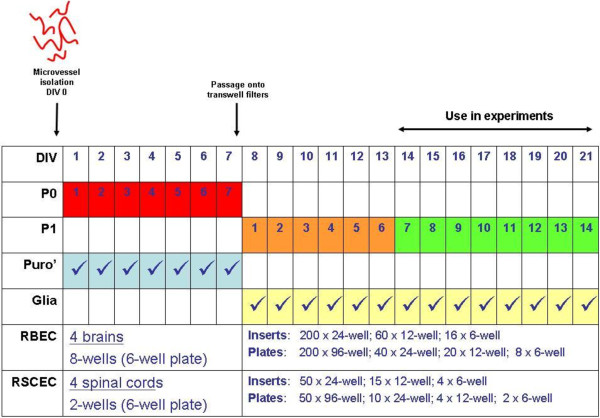
**Culture and passaging schedule for rat brain and spinal cord endothelial cells with mixed glial cells.** Maximum plating densities for cell culture inserts and tissue culture dishes are suggested for each cell type.

All procedures were carried out in accordance with the Animals (Scientific Procedures) Act, 1986. Four male Wister rats (200–250 grams, Charles River, UK) were euthanized humanely and whole brains removed and stored in HBSS buffer (calcium/magnesium-free, plus 10 mM HEPES, penicillin/streptomycin) on ice. Under aseptic conditions, the brain stem and cerebellum was dissected and each brain was cut in half sagitally. The mid-brain white matter and the choroid plexus were removed and the remaining cortical tissue rolled on dry Whatmann paper to remove the meninges. The meninges-free cortical tissue was transferred into ice-cold isolation buffer (HBSS plus calcium and magnesium, 10 mM HEPES, 0.1% BSA) and homogenised on ice using a 15 mL Dounce homogeniser with 20 strokes of the loose pestle followed by 10 strokes of the tight pestle. Following each homogenisation, the pestle was washed with isolation buffer to recover as much brain tissue as possible. The brain homogenate was pelleted by centrifugation at 240 × g for five minutes at 4°C. The supernatant was aspirated and the pellet resuspended in pre-warmed digestion mix, containing 1 mg/mL collagenase/dispase (Roche, UK), 10 μg/mL DNAse I (Roche, UK) and 0.147 μg/mL tosyl-lysine-chloromethylketone (TLCK). The tissue/digestion mix was incubated at 37°C for 30 minutes with gentle shaking. Digested tissue was pelleted by centrifugation at 240 × g for five minutes at 4°C and the pellet was resuspended in 22% (w/v) bovine serum albumin (First-Link, UK) by shaking vigorously. Centrifugation at 1500 × g for 15 minutes at 4°C resulted in a pellet containing microvessels, with a buoyant layer of myelin floating at the top. The myelin/BSA fraction was poured off, re-mixed and centrifuged again. The microvessel pellet was resuspended in isolation buffer and stored on ice. The process was repeated for a total of four centrifugations and the resuspended microvessels were pooled and pelleted by centrifugation at 240 × g for five minutes at 4°C. The supernatant was aspirated and the microvessel pellet was resuspended in pre-warmed digestion mix, followed by incubation at 37°C for 15 minutes with gentle shaking. After digestion, the microvessel fragments were pelleted by centrifugation at 240 × g for five minutes at 4°C and washed once in serum-containing cell culture medium to remove traces of enzyme. The resulting microvessel fragments were resuspended in DMEM (phenol red-free, supplemented with 15% plasma-derived serum [PDS, First-Link, UK], glutamine, BME vitamins (Sigma), antibiotic/antimycotic supplement, 200 μM ascorbic acid, 3 μM puromycin and 1× microvascular growth supplement [MVGS, Life Technologies]), and plated in eight wells over two 6-well plates pre-coated with 10 μg/cm^2^ collagen I (BD Biosciences) and 5 μg/cm^2^ fibronectin. The commercial MVGS supplement contains foetal bovine serum, hydrocortisone, human FGF, heparin, human EGF and dibutyryl cyclic AMP. Culture medium was replaced after 2–3 days *in vitro* (DIV) for identical medium, without ascorbic acid. Puromycin was maintained in the initial seven-day expansion phase of the culture to maintain selective pressure on the barrier-forming endothelial cells and to minimise the growth of any non-endothelial cells prior to passaging (Figure 1).

### Isolation of rat spinal cord endothelial cells

Spinal cords were removed from the same animals as brain tissue was taken from, and stored separately in HBSS buffer (calcium/magnesium-free, plus 10 mM HEPES, penicillin/streptomycin) on ice. Under aseptic conditions, the outer membranes were removed and the spinal cord tissue was chopped using sterile scissors into a uniform suspension. The spinal cord tissue suspension was transferred into isolation buffer on ice and pooled. Following this step, spinal cord tissue was processed in the same manner as brain tissue from the first enzymatic digestion as described above. The resulting microvessel fragments were resuspended in plating media and plated in two wells of one 6-well plate pre-coated with 10 μg/cm^2^ collagen I and 5 μg/cm^2^ fibronectin.

### Preparation of rat mixed glial feeder layer

Mixed glial cells were prepared using a protocol based on the method of McCarthy and de Vellis [66]. Ten Wistar rat pups at postnatal day 0–2 (Charles River, UK) were decapitated and whole brains removed and placed in chilled, serum-free DMEM on ice. From each brain, both cortices were removed with a sterile scalpel blade and then rolled on dry, sterile Whatmann filter paper to remove the meninges. Pooled cortical tissue was pressed through a 70 μm cell strainer (BD Falcon) to give a homogeneous cell suspension. The cell suspension was pooled and centrifuged at 240 × g for five minutes. The resulting pellet was resuspended in 100 mL of glial maintenance media (DMEM supplemented with 10% FBS, glutamine and penicillin/streptomycin) and plated out into 10 × T75 flasks, 10 mL per flask. The cell suspension was cultured undisturbed for 1 week (37°C, 5% CO_2_) before a full media change to remove non-adherent cell debris. The proliferating mixed glial preparation was cultured for a further 14 days to allow growth, with a full media change after 7 days. After a total of 21 days in culture, the mixed glia were passaged with 0.25% trypsin:EDTA, resuspended in astrocyte freezing medium (DMEM, 10% FBS, 10% DMSO) at a concentration of 2–3 million cells/mL and frozen at a rate of 1°C/min at −80°C using a Nalgene freezing container. For establishing mixed glial feeder layers for co-culture with endothelial cells, single vials of frozen mixed glia were quickly thawed in a 37°C water bath and added drop-wise to 50 mL pre-warmed (37°C) glial maintenance media. Cells were pelleted at 240 × g for five minutes and resuspended in 5 mL glial maintenance media before plating in a single T25 flask. Mixed glial cells were grown to 95% confluence before passage with 0.25% trypsin: EDTA and seeding into 24-well plates at least 3 days before cell culture inserts with endothelial cells were added to the 24-well plate.

### Passage of primary rat brain and spinal cord endothelial cells onto cell culture inserts and tissue culture plates

Rat brain or spinal cord endothelial cells grown on collagen I/fibronectin-coated plates were passaged at ~95% confluence. Cells were washed twice with pre-warmed PBS and 400 μL of pre-warmed 0.25% trypsin was added to each well. The plates were immediately returned to a 37°C incubator for four minutes. The trypsinisation was stopped by adding 1 mL of cell culture medium containing 15% PDS to each well. The endothelial cells were washed off and resuspended by gently pipetting up and down. Cells were split at a ~1:1 ratio, on a surface area basis. For example, the 9.5 cm^2^ of a single well on a 6-well plate could cover the equivalent of 28 × 24-well cell culture inserts each with a surface area of 0.33 cm^2^ (Figure 1). Cells were resuspended in either DMEM/MVGS (phenol red-free DMEM supplemented with 20% PDS, glutamine, BME vitamins, antibiotic/antimycotic supplement and 1× MVGS) or EBM-2/EGM-2 (EBM-2 media plus 15% PDS, glutamine, BME vitamins, BulletKit SingleQuots minus the human recombinant VEGF supplement [recombinant human FGF, recombinant human EGF, recombinant human IGF, hydrocortisone, GA-1000, ascorbic acid], all Lonza, UK) media formulations. The MVGS supplement does not contain VEGF, a factor known to increase permeability across brain endothelial cell monolayers [52], and so this factor was not added from the EGM-2 BulletKit. The resuspended cells at the adjusted concentration were plated in the upper chambers of cell culture inserts in the 24-well format at 200 μL/well (Millipore, PET, 1.0 μm pore size). Pre-seeded mixed glial cells were switched from astrocyte maintenance medium into 1 mL DMEM/MVGS or EBM-2/EGM-2 and the inserts with endothelial cells were added. Brain and spinal cord endothelial cells and astrocytes were cultured for a further 7–14 days, with media changes every 2–3 days.

### Measurement of transendothelial electrical resistance

Cells cultured on inserts in 24-well plates were removed from the tissue culture incubator (37°C, 5% CO_2_), and allowed to equilibrate to room temperature for 20 minutes. TEER values were measured using an EVOM2 voltometer with STX-2 electrodes (World Precision Instruments). To calculate TEER (Ω (Ohms) × cm^2^), electrical resistance across a collagen I/fibronectin-coated insert without cells was subtracted from the readings obtained on inserts with cells and this value was multiplied by the surface area of the insert (0.33 cm^2^).

### Monolayer permeability to Lucifer yellow/FITC-labelled dextrans and calculation of permeability coefficients

Lucifer yellow (LY) and FITC-labelled dextran stock solutions were prepared in Ringers-HEPES buffer (150 mM NaCl, 3.4 mM CaCl_2_, 1.2 mM MgCl_2_, 5.2 mM KCl, 0.5 mM NaHCO_3_, 2.8 mM glucose, and 10 mM HEPES) and frozen at −20°C. RBECs were passaged onto cell culture inserts as described and cultured for a further 7–14 days *in vitro*. For transport experiments, all media was removed from the upper chamber of the insert and replaced with 75 μL pre-warmed Ringers-HEPES buffer plus 0.1% BSA followed by equilibration to 37°C for 10–15 minutes. Solutions of LY and FITC-labelled dextran were diluted to 2X working concentrations and pre-warmed to 37°C. At time-point 0 minutes, 75 μL of LY/FITC-labelled dextran solution was added to the upper chamber of the inserts, which were then transferred to new 24-well plates containing 1 mL of pre-warmed Ringers-HEPES buffer plus 0.1% BSA. For each compound, inserts with endothelial cells were used in triplicate and cell-free, collagen I/fibronectin-coated inserts were used in duplicate. The plates were incubated in an orbital shaking incubator (VWR) at 37°C, 25 rpm. At each time-point, the inserts were moved into a fresh 24-well plate containing 1 mL of pre-warmed Ringers-HEPES buffer plus 0.1% BSA, to prevent back-diffusion of the compounds into the top chamber. Samples were collected at 30, 60 and 90 minutes. At the end of each experiment, the concentration of the fluorescent compounds accumulated in the bottom chamber was calculated by transferring 50 μL of each sample to a black walled-96 well plate (Nunc) and measuring with an Envision fluorescence plate reader (Perkin Elmer). Concentrations were calculated using standard curves generated from the stock solutions of each compound. Permeability coefficients (Pe), that take into account the barrier to transport from both the endothelial monolayer and the cell culture insert, were calculated as described by others [11,31,67,68]. Briefly, the volume cleared across cell-free and cell-containing inserts was calculated for each compound using the following equation:

$$\text{Cleared}\;\text{volume}\;\left(\mathrm{\mu L}\right)=\frac{{\mathrm{Concentration}}_{\text{abluminal}}\times {\text{volume}}_{\text{abluminal}}}{{\text{Concentration}}_{\text{luminal}}}$$

The average cleared volumes were plotted versus time in minutes for each 90-minute experiment. Clearance slopes for the empty filters (PS_filter_) and the filters with endothelial cells (PS_cells + filter_) were calculated using linear regression analysis and used to obtain a permeability product value for endothelial monolayer alone (PS_cells_):

$$\frac{1}{{\text{PS}}_{\text{cells}}}=\frac{1}{{\mathrm{PS}}_{\text{cells}+\text{filter}}}-\frac{1}{{\mathrm{PS}}_{\text{filter}}}$$

Permeability coefficients (Pe) for each compound across the cell monolayer were finally derived by dividing the PS_cells_ value by the surface area of the cell culture insert (0.33 cm^2^ for 24-well format). Data are presented with units of × 10^-6^ cm/sec.

### Assessment of claudin-5 protein levels by Western blotting

RBECs were passaged into two 35 mm dishes, one with RBEC/MVGS formulation media and one with EBM-2/EGM-2 formulation media. The cells were cultured to confluence and then lysed on ice by the addition of RIPA buffer (Sigma) with protease inhibitors followed by scraping. The levels of claudin-5 protein present in 10 μg total soluble protein were assessed by SDS-PAGE and Western blotting using the mouse anti-claudin-5 antibody (at 1:500; 1 μg/mL) also used for immunocytochemistry (Table 1). The membranes were re-probed using a mouse monoclonal antibody (ACTN05 (C4), Abcam, 1:2000). Western blots were imaged using a Li-Cor Odyssey CLx and quantification of band intensity was carried out using the Li-Cor software.

**Table 1:** Antibodies used for immunocytochemical characterisation of cultured RBECs and RSCECs

**Antigen**	**Species**	**Manufacturer**	**Concentration**
Caveolin 1	Rabbit	Abcam ab2910	20 μg/ml
Clathrin heavy chain	Rabbit	Abcam ab21679	20 μg/ml
Claudin-5	Mouse	Life Technologies 35-2500	10 μg/ml
Occludin	Mouse	Life Technologies 33-1500	10 μg/ml
P-gp	Mouse	Abcam ab3366	3.35 μg/ml
Smooth muscle actin	Mouse	R & D Systems MAB1420	4 μg/ml
Von Willebrand factor	Rabbit	Abcam ab6994	156 μg/ml
ZO-1	Rabbit	Abcam ab59720	10 μg/ml

### Immunocytochemistry

Immunocytochemistry was performed on RBECs and RSCECs cultured on collagen I/fibronectin coated 96-well plates. Cells were cultured to confluence and maintained for a further two days. Cultures were fixed in either ice-cold methanol for two minutes (antibodies for tight junction protein staining) or in 3.7% formaldehyde for 20 minutes at room temperature (all other antibodies). Formaldehyde-fixed cells were permeabilised with 0.2% Triton X-100 in PBS for five minutes. After rinsing once in PBS, cells were blocked in 5% BSA in PBS for 30 minutes. All antibodies were diluted to working concentration in 1% BSA in PBS (Table 1). Cells were incubated with primary antibody for 1 hour at room temperature or overnight at 4°C, followed by three 5 minute washes in PBS. Secondary antibodies (Alexa Fluor 488 donkey anti-goat IgG, Alexa Fluor 488 goat anti-mouse IgG, Alexa Fluor 546 goat anti-mouse IgG, Alexa Fluor 488 goat anti-rabbit IgG, Alexa Fluor 546 donkey anti-rabbit IgG; all from Life Technologies, Molecular Probes) were used at a final concentration of 2 μg/mL. Cells were incubated with secondary antibody for 1 hour at room temperature followed by three 5 minute washes in PBS. Cells were finally counter-stained with Hoechst (Life Technologies, Molecular Probes), diluted to 1 μg/mL in 1% BSA/PBS, for one minute and rinsed a further three times in PBS. Samples were imaged using an Olympus IX81 fluorescence microscope.

### Analysis of small molecule permeability using liquid chromatography/mass spectrometry

Small molecule compounds were dissolved in DMSO to a concentration of 1 mM and further diluted in Ringers-HEPES buffer (without BSA) to give a final concentration of 4 μM. RBECs and RSCECs were passaged onto cell culture inserts as described and cultured for a further 7–14 days *in vitro*. Cell culture medium (EBM-2/EGM-2 formulation) was removed from the upper and lower compartments of RBECs and RSCECs cultured in triplicate on cell culture inserts and duplicate cell-free inserts and replaced with Ringer-HEPES buffer (without BSA). The small molecules were added to each upper compartment to yield a final concentration of 2 μM. Cultures were incubated at 37°C with shaking and transferred to a new well with fresh buffer in the lower compartment after 30, 60 and 90 minutes. Samples were collected from the lower compartments and analysed by liquid chromatography mass spectrometry (LC-MS/MS). Small molecules were analysed on an Acquity™ UPLC system with an Acquity UPLC_®_ BEH C18, 1.7 μm column (Waters Corp., Milford, MA, USA). 10 μL of each sample was injected onto the column and eluted by gradients. The flow rate was 0.6 mL/min and the run time was 1.1 min. The Acquity™ UPLC-system was connected to a triple quadrupole tandem mass spectrometer (Quattro Premier XE, Waters Corp., Milford, MA, USA) operating in the positive ion electrospray ionisation mode, with MassLynx 4.1 running in the MRM mode (MS/MS). Permeability coefficients were calculated as described above.

### P-gp functional efflux assay

RBECs and RSCECs were passaged onto cell culture inserts as described and cultured for a further 7–14 days *in vitro*. Cell culture medium (EBM-2/EGM-2 formulation) was removed from the upper and lower compartments of RBECs and RSCECs cultured on cell culture inserts in 24-well plates and was replaced with Ringers-HEPES buffer with 0.1% BSA, containing either 100 μM verapamil or vehicle (0.5% DMSO), followed by incubation at 37°C for 30 minutes. Cells were dye-loaded by removing buffer from upper compartments and replacing with fresh buffer containing 200 ng/ml rhodamine 123. Triplicate cell culture inserts with cells were used for each condition. The inserts were incubated at 37°C for 30 minutes. The dye-loaded inserts were transferred to a fresh plate and the buffer was removed from the upper compartments. The cells were washed three times in Ringer-HEPES buffer (with 0.1% BSA). Fresh assay buffer was added and the inserts were incubated at 37°C for 1 hour to allow dye efflux. At the end of the incubation, the inserts were transferred to a fresh plate, the cells were washed three times in PBS and lysed for 20 minutes in RIPA buffer. Fluorescence values were measured for each sample using an Envision multi-well fluorescence plate-reader (Perkin Elmer) with excitation at 485 nm and emission collected at 535 nm. Standard curves were generated using stock rhodamine 123 and then used to calculate cellular uptake of the dye.

### Determination of FITC-dextran hydrodynamic radii by dynamic light scattering

FITC-labelled dextrans (Sigma) were prepared at a concentration of 0.8 mg/mL in Ringers-HEPES buffer without BSA. Samples were filtered through a 0.22 μm filter prior to loading. Hydrodynamic radii were determined using a Zetasizer Nano (Malvern). The backscatter of light at 173° was measured with an equilibration time of five minutes and measurements were performed in triplicate with no delay between them. Laser attenuation and measurement duration were determined automatically by the software with data processing performed at normal resolution.

### Analysis and statistics

Standard curves were generated and sample concentrations interpolated by linear regression using Microsoft Excel. Statistical analysis, using the appropriate mathematical functions as outlined in the text, was carried out using GraphPad Prism. Values in figures are expressed as mean ± SEM.

## Results

### Isolation and culture of microvascular endothelial cells from rat brain and spinal cord tissue

**Figure 2:**
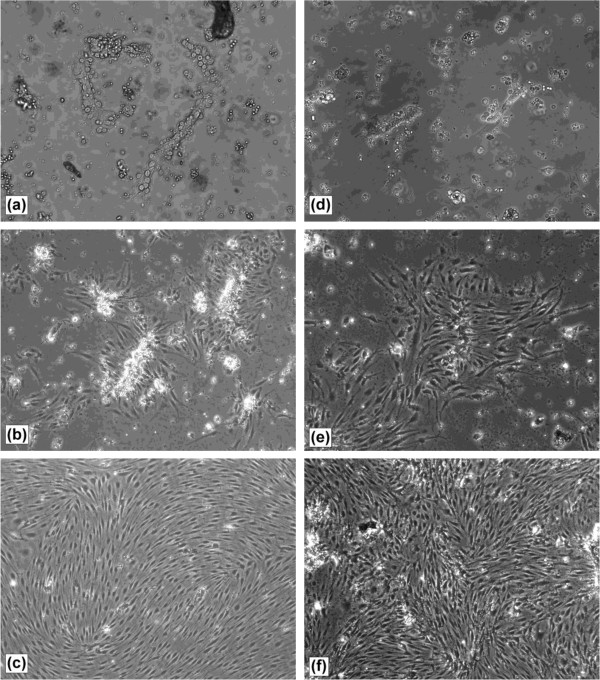
**Isolation and culture of rat brain and spinal cord microvascular endothelial cells.** Following BSA density centrifugation and enzymatic digestion, isolated rat brain and spinal cord microvessel fragments were plated out onto collagen 1/fibronectin coated tissue culture plates. On plating, (**a**) brain and (**d**) spinal cord microvessels exhibit a “beads-on-string” appearance with rounded endothelial cells present on the surface (20× objective magnification). After 2–3 days in culture, (**b**) brain and (**e**) spinal cord endothelial cells are clearly visible migrating from the microvessels onto the matrix-coated tissue culture dish (10× objective magnification). After 5–7 days in culture both (**c**) brain and (**f**) spinal cord endothelial cells form a pure, near confluent monolayer (10× objective magnification).

To establish a convenient cell culture system that generated a large yield of cells with minimum use of animal tissue, microvascular endothelial cells were isolated from the brain and spinal cord tissue of the same four rats. We took the approach of plating isolated microvessels into 6-well tissue culture dishes and allowing endothelial cells to grow to near (>90%) confluence before passaging on to cell culture inserts. To obtain the highest recovery of microvessels, brain and spinal cord homogenates were centrifuged through a 22% BSA gradient to obtain a vasculature pellet. We did not subject the brain and spinal cord vasculature pellets to a size dependent filtration step, as this resulted in the loss of some microvessel fragments, thus decreasing the final yield of endothelial cells. Following enzymatic digestion with collagenase/dispase, brain and spinal cord vasculature fragments exhibited typical “beads-on-string” appearance (Figure 2a, d) as described by others [20]. We routinely cultured the brain and spinal cord microvessel fragments in DMEM with a commercially available microvascular growth supplement (MVGS, Life Technologies) and 15% plasma-derived serum (PDS), to minimise the PDGF-stimulated growth of non-endothelial cells [31,61]. Upon plating, microvessel fragments adhered to the extracellular matrix coated dishes within 1–2 hours. The largest and most branched sections of the vasculature did not adhere to the plate and were easily removed during media changes. Under these conditions, rat brain endothelial cells (RBECs) and rat spinal cord endothelial cells (RSCECs) migrated out from the isolated fragments and proliferated rapidly, reaching near confluence after 6–7 days (Figures 1 and 2a-f). When culturing from four adult rats into a 6-well plate format, a total of eight wells of RBECs and two wells of RSCECs could be established, ready for passage within 6–7 days (Figure 1). During the initial plating phase, we adopted the technique of culturing isolated microvessels in the presence of puromycin to limit the growth of non-endothelial cells lacking expression of the P-gp efflux transporter [22,27,31,61,65]. Inspection of the RBEC and RSCEC cultures by phase contrast microscopy indicated that they were near-pure endothelial monolayers (Figure 2c, f).

### Passaging technique and characterisation of barrier formation using rat blood–brain barrier endothelial cells on cell culture inserts

Once brain and spinal cord endothelial cells could be reproducibly isolated and cultured from the same rats, we investigated passaging techniques onto cell culture inserts and tissue culture dishes to utilise the large number of cells generated. As more endothelial cells were obtained from rat brain tissue compared to spinal cord, we optimised our passaging and culture conditions using RBECs. Near confluent monolayers of RBECs at 6–7 days in culture were passaged with trypsin onto cell culture inserts. We found it better to use a relatively high concentration of trypsin: EDTA (0.25%) for a short amount of time (3–4 minutes), rather than lower concentrations for a longer time period. Milder passaging reagents, such as Accutase™ did not effectively remove the primary endothelial cells from the culture dish, nor break down junctions between cells. The most likely reason for these observations was that the endothelial cells already possessed strong intercellular tight junctions. We thus trypsinised and dissociated the primary monolayers to small clusters of approximately 5–10 endothelial cells. Confluent monolayers were not reproducibly obtained when performing passages that diluted the cell suspension of RBECs 1:2 to 1:4-fold. We were, however, able to obtain reproducible confluence when the trypsinised RBEC cell suspension was transferred ~1:1 on a surface area basis; for example plating one well from the 6-well plate into 25 cell culture inserts in the 24-well format (Figure 1). This passaging method allowed quick coverage of the surface area of the insert, and the cells were able to reproducibly form barriers.

We investigated whether commercially available speciality endothelial cell culture reagents could influence both the quantity of endothelial cells recovered and the quality of the rat *in vitro* barriers generated by this passaging technique. Following initial plating in DMEM with MVGS supplement, we passaged the RBECs onto collagen I/fibronectin-coated cell culture inserts and compared two media formulations in both the top well and bottom well: (a) DMEM with MVGS, and (b) EBM-2 microvascular endothelial cell media with the EGM-2 BulletKit without VEGF (Lonza). The endothelial cells were co-cultured with mixed glia plated into the bottom chamber of the dish, as the role of astrocytes in inducing barrier phenotype in primary brain endothelial cells *in vitro* is well validated [7,42,69,70]. We quantified barrier phenotype by two standard methods; TEER, measured at room temperature, and paracellular permeability to Lucifer yellow over 90 minutes. When measuring TEER, we took the approach of removing the cells from the incubator and allowing them to equilibrate to room temperature. This technique allowed greater consistency in TEER readings when measuring with the commonly-used STX2 chop-stick electrodes. Measuring large numbers of inserts directly after removal from the incubator resulted in erroneous measurements due to media buffering when moving from the regulated temperature and CO_2_ of a tissue culture incubator. When removing inserts and measuring resistance immediately, we found that TEER rose steadily until a stable level was reached after approximately 20 minutes. Allowing TEER values to stabilise at room temperature increased the accuracy and consistency of the measurements when measuring a large number of inserts.

**Figure 3:**
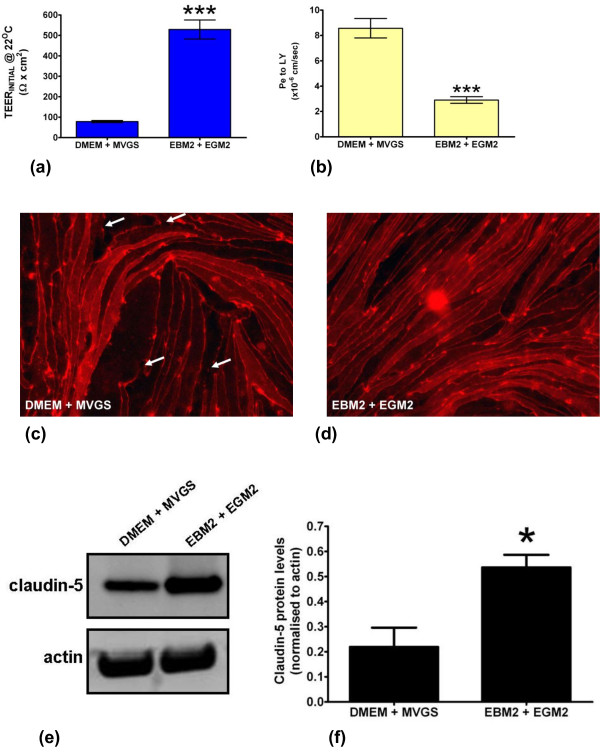
**Effect of media composition on RBEC barrier formation and characteristics.** (**a**) Comparison of the effects of the DMEM/MVGS and EBM-2/EGM-2 media formulations on the TEER of RBECs grown for 14 days on cell culture inserts. Data is presented as mean ± SEM and was analysed using an unpaired, two-tailed students *t*-test, ***P < 0.0001; n = 5 independent cell culture experiments in 24-well plates, with 3 inserts per experiment, equivalent to 15 inserts total . (**b**) Calculated permeability coefficients for the paracellular passage of 100 μM (50 μg/mL) Lucifer yellow over a 90 minute period at 37°C across RBEC monolayers on cell culture inserts cultured in DMEM/MVGS and EBM-2/EGM-2 media formulations. Data is presented as mean ± SEM and was analysed using an unpaired, two-tailed students *t*-test, ***P < 0.0001; n = 5 independent cell culture experiments, with 3 inserts per experiment, equivalent to 15 inserts total. Fluorescence microscope images of RBECs stained with an antibody raised against the tight junction protein claudin-5 following culture in (**c**) DMEM/MVGS supplement, or (**d**) EBM-2/EGM-2. White arrows indicate regions of discontinuous claudin-5 staining. Images are representative of 3 independent cultures, with five fields of view taken from each individual preparation of cells using the 20× objective on an Olympus IX81 microscope. (**e**) Western blot analysis of claudin-5 protein expression levels in RBECs cultured in DMEM/MVGS and EBM-2/EGM-2. Blots were reprobed with anti-actin antibodies as a control for equal loading of cell lysates. (**f**) Densitometry analysis of claudin-5 band intensity, normalised to actin levels, for RBECs grown in DMEM/MVGS vs. EBM-2/EGM-2. Data is presented as mean ± SEM and was analysed using an unpaired, two-tailed students *t*-test, *P < 0.01; n = 3 independent experiments.

Both the DMEM/MVGS and EBM-2/EGM-2 media formulations lead to the development of reproducibly robust barriers after 14 days in culture (Figure 3a, b). Average pre-experimental TEER values were significantly higher for the RBECs cultured in the EBM-2/EGM-2 media when compared to the DMEM/MVGS formulation, with average TEER values at room temperature of 529 ± 14 Ω × cm^2^ versus 90 ± 3.6 Ω × cm^2^ (Figure 3a). Peak TEER values measured at room temperature in this experiment were as high as 802 Ω × cm^2^ in EBM-2/EGM-2, versus 252 Ω × cm^2^ for the DMEM/MVGS formulation. In agreement with the TEER data, small molecule permeability for the same cell cultures was significantly decreased for the passaged RBECs cultured in EBM-2/EGM-2 media, with permeability coefficients averaging 2.9 ± 0.26 × 10^-6^ cm/sec compared to 8.6 ± 0.76 × 10^-6^ cm/sec for DMEM/MVGS (Figure 3b). To explore the effect of the two media conditions on tight junction formation we immunostained cells grown on cell culture inserts with an antibody raised against claudin-5, a protein whose role in establishing restrictive barrier phenotype in brain endothelial cells is well documented *in vivo* and *in vitro*[71-73]. The RBECs grown in DMEM/MVGS showed localisation of claudin-5 around the periphery of the cells, indicating intercellular tight junction formation (Figure 3c). Under these conditions however, several areas of discontinuous staining were also observed, indicating potentially “leaky” gaps in the endothelial tight junctions (Figure 3c). RBECs grown in the EBM-2/EGM-2 media formulation however, showed increased cell density and continuous claudin-5 staining at the cell periphery, suggesting the formation of highly organised, continuous tight junctions (Figure 3d). Western blot analysis of cell lysates prepared from RBECs cultured in the two different conditions, demonstrated that the overall expression of claudin-5 was significantly increased in the EBM-2/EGM-2 conditions, with a 2.4-fold increase in protein levels (Figure 3e, f), The difference in claudin-5 expression and localisation at tight junctions observed between cells cultured in the two media formulations may contribute to the higher TEER and lower Pe to LY observed when culturing RBECs in EBM-2/EGM-2. Culturing passaged primary RBECs in the endothelial EBM-2/EGM-2 media combination thus significantly improved the quality of the barrier phenotype developed by these high yield cell cultures.

**Figure 4:**
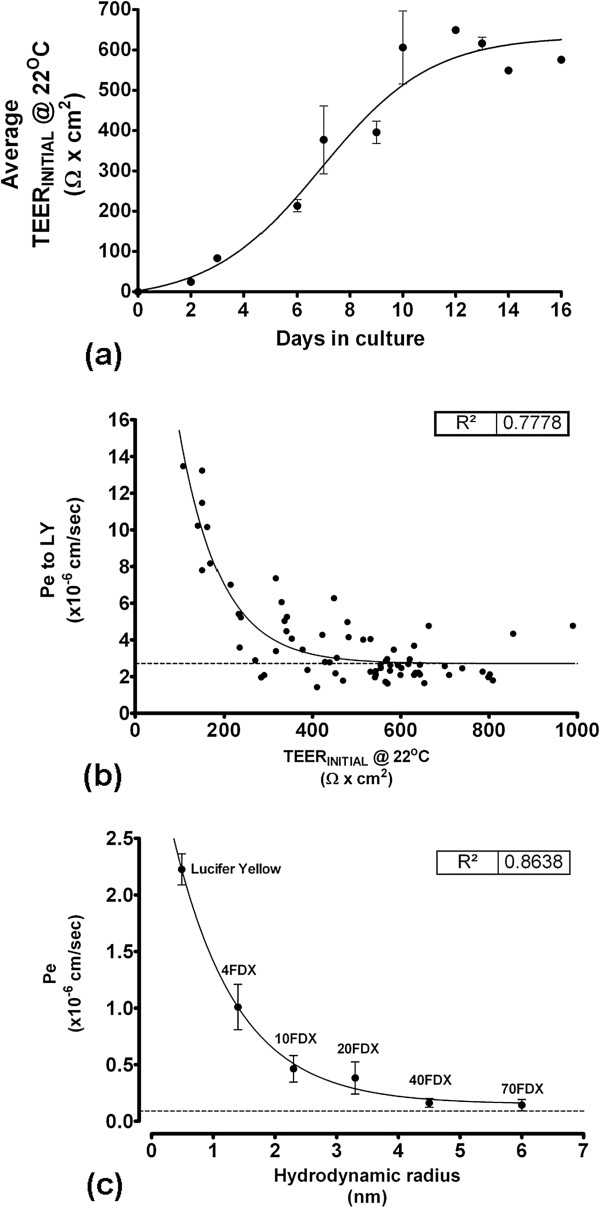
**Characterisation of RBEC monolayer barrier function induced by co-culture in optimised EBM-2/EGM-2 media.** (**a**) Development and stabilisation of TEER for passaged RBECs grown on cell culture inserts in the optimised EBM-2/EGM-2 conditions over a two week period. TEER was measured following equilibration of the inserts in cell culture medium to room temperature, n = 5 independent cell culture experiments. (**b**) Relationship between pre-experimental TEER and permeability to Lucifer yellow over 90 minutes at 37°C in the EBM-2/EGM-2 media conditions. Data was fitted to a one-phase exponential decay curve, R^2^ = 0.78, n = 6 independent cell culture experiments, equivalent to 71 inserts in total. Peak TEER at room temperature in this experiment reached as high as 999 Ω × cm^2^_._ (**c**) Permeability of Lucifer yellow (50 μg/mL) and FITC-labelled dextrans (10 mg/mL) of increasing hydrodynamic radius across RBEC monolayers in the optimised EBM-2/EGM-2 media conditions. Pe was calculated over a 90 minute time-course and was plotted versus the hydrodynamic radius of each molecule. Data was fitted to a one-phase exponential decay curve, R^2^ = 0.86, n = 2 independent cell culture experiments, equivalent to 6 inserts in total for each molecule tested.

We further characterised RBEC barrier function in the optimal EBM-2/EGM-2 culture conditions. TEER values in the optimised media reached a maximum at 10 days in culture and remained at this level for several days, indicating the persistent formation of continuous tight junctions (Figure 4a). Furthermore, for RBECs passaged onto cell culture inserts in the optimised conditions, a strong correlative relationship was observed between the pre-experimental TEER values and subsequent permeability to Lucifer yellow (Figure 4b). This relationship fitted an exponential decay curve (R^2^ = 0.78), indicating that as TEER decreased, Pe to LY markedly increased. The exponential decay curve reached plateau at an equivalent Pe to LY of 2.7 × 10^-6^ cm/sec. Such an exponential relationship in endothelial permeability is in accordance with previously described data in primary brain endothelial cells from other species [74] and in brain endothelial cell lines [75].

We next characterised RBEC barrier permeability to larger molecules that non-specifically cross the monolayer by paracellular diffusion. RBECs cultured on cell culture inserts in EBM-2/EGM-2 media were used to measure the permeability coefficients for FITC-labelled dextrans of increasing size (Figure 4c). The observed Pe value for transport of each FITC-labelled dextran molecule was plotted versus its hydrodynamic radius (HR) (Figure 4c). When performing such experiments it is more accurate to use the HR of a molecule rather than its molecular weight. Molecules of the same weight can have different HR and diffusion profiles in solution due to their shape (e.g. rod-like FITC- labelled dextrans versus spherical globular proteins). To obtain accurate hydrodynamic radii for the FITC-labelled dextrans used, we analysed each molecule using dynamic light scattering (DLS, Table 2). When permeability was plotted versus HR, a strong relationship between the two was observed, which fitted to an exponential decay curve (R^2^ = 0.86, Figure 4c). The smaller molecules showed the highest non-specific paracellular permeability, and permeability reached plateau at Pe = 0.09 × 10^-6^ cm/sec, corresponding to molecules with a hydrodynamic radius of 4.5 nm (i.e. 40 kDa FITC-dextran) and above.

**Table 2:** Experimentally determined hydrodynamic radii and observed permeability coefficients across RBEC monolayers for Lucifer yellow and FITC-dextrans

**Molecule**	**Molecular weight (kDa)**	**Hydrodynamic radius (nm)**	**Mean paracellular transport (× 10**^**-6**^ **cm/sec)**	**SEM (× 10**^**-6**^ **cm/sec)**
Lucifer Yellow	0.5	0.49	2.22	0.137
4 kDa FITC-Dextran	4	1.40	1.00	0.201
10 kDa FITC-Dextran	10	2.30	0.46	0.117
20 kDa FITC-Dextran	20	3.30	0.38	0.142
40 kDa FITC-Dextran	40	4.50	0.16	0.038
70 kDa FITC-Dextran	70	6.00	0.14	0.049

### Characterisation of barrier-related protein expression in rat brain endothelial cells cultured in EBM-2/EGM-2 media

**Figure 5:**
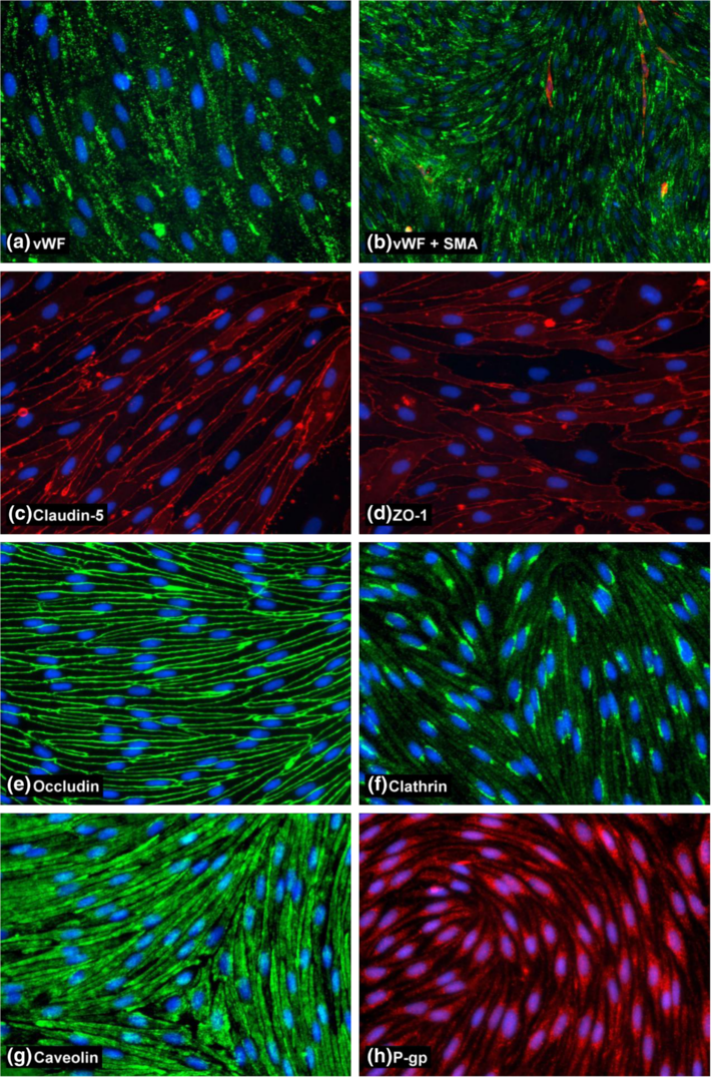
**Characterisation of BBB protein expression in cultured RBECs by immunocytochemistry.** Images show fixed and permeabilised RBECs cells grown on collagen I and fibronectin-coated 96-well plates and stained with antibodies for (**a**) von Willebrand Factor, (**b**) smooth muscle actin, (**c**) claudin-5, (**d**) ZO-1, (**e**) occludin, (**f**) clathrin heavy chain, (**g**) caveolin-1, (**h**) P-gp. Images are representative of 3 independent cultures, with five fields of view taken from each individual preparation of cells using the 20× objective [10× for 5 (b) for a wider field of view] on an Olympus IX81 microscope.

Having established that the most reproducible and robust RBEC barrier phenotypes were induced by co-culture in EBM-2/EGM-2 media, we performed a more extensive characterisation of the cells using immunocytochemistry (Table 1, Figure 5). The passaged RBEC monolayers were found to be essentially purely endothelial in nature as judged by antibody staining with the endothelial marker von Willebrand factor (vWF, Figure 5a). There were no non-endothelial cells disrupting the monolayer and any such cells present in the passaged cultures were most likely to be smooth muscle actin (SMA)-positive pericytes sitting on top of the endothelial monolayer without perturbing its confluence (Figure 5b). Staining with antibodies recognising the tight junction markers claudin-5, ZO-1 and occludin, all well-known to be required for BBB function, showed the presence of continuous and well-organised tight junctions between cells in the endothelial monolayers (Figure 5c, d, e). Some diffuse cytoplasmic staining of claudin-5 and ZO-1 was observed, but all endothelial cells presented highly organised tight junction staining without gaps or frayed edges. We also investigated two major endocytic/transcytotic pathways in the cultured RBECs by performing immunocytochemistry with antibodies specific for clathrin and caveolin-1. RBECs showed strong immunoreactivity for both clathrin (Figure 5f) and caveolin (Figure 5g). Caveolin-1 was distributed evenly and densely throughout the cytoplasm of the RBECs, with individual puncta difficult to observe. In contrast, clathrin staining was more punctuate in nature, with most RBECs showing a distinct concentration of clathrin-positive puncta in a peri-nuclear location. Staining with antibodies raised against the P-gp efflux transporter, revealed expression of this important transporter in the cultured RBECs (Figure 5h). These immunocytochemistry data thus support our previous observations of a tight barrier phenotype of pure endothelial cells with limited paracellular permeability.

### Establishment and characterisation of rat blood-spinal cord endothelial *in vitro* barriers

**Figure 6:**
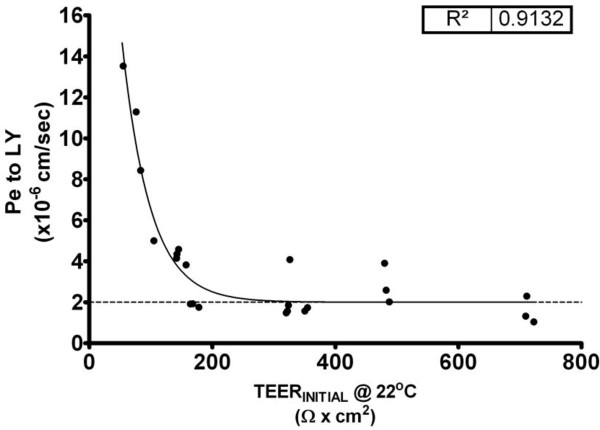
**Barrier function demonstrated by RSCEC monolayers co-cultured on cell culture inserts with mixed glial cells.** Relationship between pre-experimental TEER, measured at room temperature, and permeability to Lucifer yellow over 90 minutes at 37°C in the optimised EBM-2/EGM-2 media conditions. Data was fitted to a one-phase exponential decay curve, R^2^ = 0.91, n = 8 independent cell culture experiments, 24 cell culture inserts in total.

Having successfully established a protocol for passaging RBECs to increase the cell yield and induce reproducibly tight barrier phenotypes, we analysed whether the optimised EBM-2/EGM-2 media would have similar effects on the endothelial cells that were isolated from rat spinal cord (Figure 1). We isolated and cultured primary RSCECs and passaged the cells in a similar manner to the RBECs, splitting at a ~1:1 transfer ratio onto cell culture inserts. The passaged RSCECs were then cultured in the presence of pre-seeded mixed glia in 24-well plate format, using the EBM-2/EGM-2 media formulation supplemented with 15% PDS. The RSCECs exhibited tight barrier characteristics with average pre-experimental TEER values of 293 ± 43 Ω × cm^2^ and Pe to LY of 3.8 ±0.67 × 10^-6^ cm/sec (Table 3). Individual pre-experimental TEER values at room temperature reached values as high as 722 Ω × cm^2^_,_ with Pe to LY values as low as 1.02 × 10^-6^ cm/sec. Much like the barriers formed by the passaged RBECS in the optimised media conditions, the RSCECs showed an exponential decay relationship between the pre-experimental TEER value recorded at room temperature and the permeability to Lucifer yellow (Figure 6, R^2^ = 0.91). The exponential decay curve reached plateau at an equivalent Pe to LY of 0.28 × 10^-6^ cm/sec. Thus, although the RSCECs did not reach overall pre-experimental TEER values as high as those observed for cultured RBECS in the optimised culture conditions, they showed similarly low paracellular permeability to small molecules such as LY.

**Table 3:** **Comparison of the *in vitro *barrier tightness of RBEC and RSCEC monolayers cultured on Millipore cell culture inserts**

	**Ave. pre-experimental TEER ± SEM (Ohms** × **cm**^**2**^**)**	**Ave Pe to LY ± SEM (cm/sec)**	**Maximum observed pre-experimental TEER (Ohms × cm**^**2**^**)**	**Lowest Pe to LY (cm/sec)**	**R**^**2**^**(TEER vs. Pe to LY)**
RBEC	529 ± 14	2.9 ± 0.26 × 10^-6^	999	1.4 × 10^-6^	0.78
RSCEC	293 ± 0.26	3.8 ± 0.67 × 10^-6^	722	1.04 × 10^-6^	0.91

**Figure 7:**
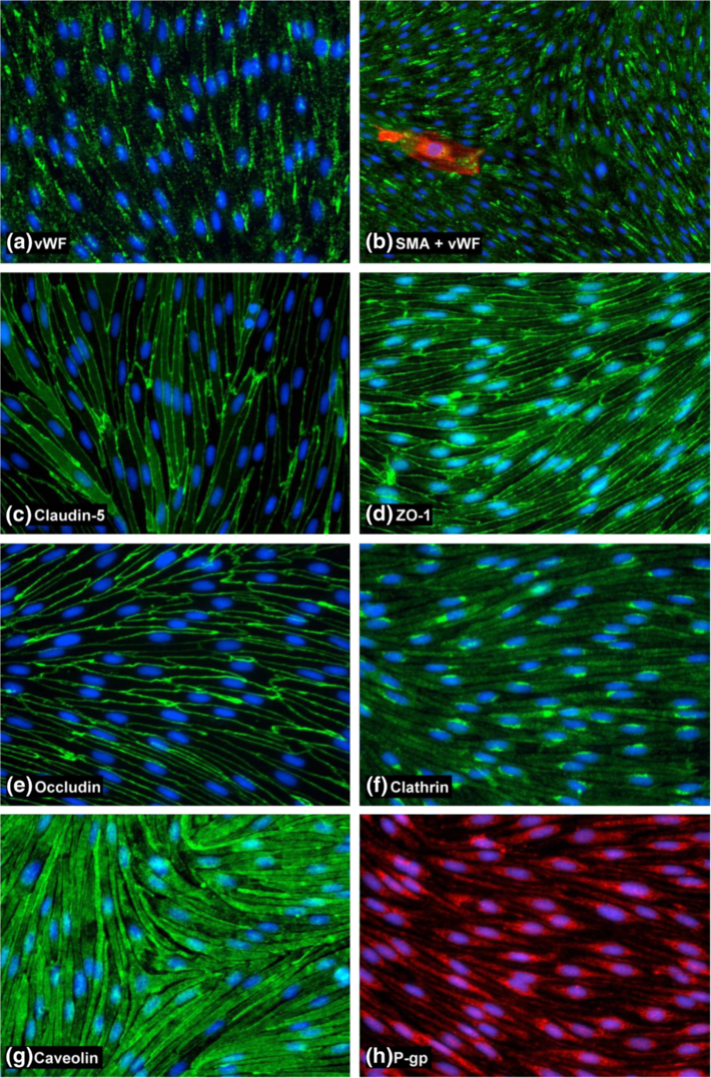
**Characterisation of cultured RSCECs by immunocytochemistry.** Images show fixed and permeabilised RSCECs cells grown on collagen I and fibronectin-coated 96-well plates and stained with (**a**) von Willebrand Factor, (**b**) smooth muscle actin and vWF, (**c**) claudin-5, (**d**) ZO-1, (**e**) occludin, (**f**) clathrin heavy chain, (**g**) caveolin, (**h**) P-gp. Images are representative of 3 independent cultures, with five fields of view taken from each individual preparation of cells using the 20× objective [10× for 7(b) for a wider field of view] on an Olympus IX81 microscope.

The passaged RSCECs were then cultured in the optimised EBM-2/EGM-2 media conditions and characterised by immunocytochemistry, using the same panel of antibodies as for the RBEC characterisation (Table 1, Figure 7). Similar to the RBEC cultures, the RSCECs were found to be essentially pure preparations of endothelial cells, exhibiting uniform staining with an antibody raised against the endothelial marker vWF (Figure 7a). Only isolated cells on top of the continuous monolayer stained positive for the non-endothelial marker SMA, making up less than 1% of the cultures (Figure 7b). The RSCECs exhibited highly organised tight junctions and claudin-5, occludin and ZO-1 were all detected with discrete, continuous localisation around the periphery of the endothelial cells with no frayed edges (Figure 7c, d and e). Immunostaining with antibodies raised against clathrin and caveolin-1 demonstrated that the RSCECs expressed these proteins in a similar pattern to the RBECs (Figure 7f, g). Caveolin-1 was detected throughout the cell cytoplasm, stopping just short of the cell edges, whereas clathrin was more punctuate and concentrated in a peri-nuclear region. These results indicate that the cultured RSCECs have the relevant protein machinery in place for two of the major endocytic and trafficking pathways in endothelial cells when cultured *in vitro*. Expression of P-pg efflux transporter was also confirmed in the cultured RSCEC cells (Figure 7h). Our observations thus demonstrate that pure RSCECs co-cultured in the optimised EBM-2/EGM-2 media conditions showed tight barrier function and expression of endothelial markers, tight junction proteins, endocytosis machinery and P-gp efflux transporter when cultured *in vitro*.

### Utility of optimised RBEC and RSCEC monolayers for small molecule drug discovery

**Figure 8:**
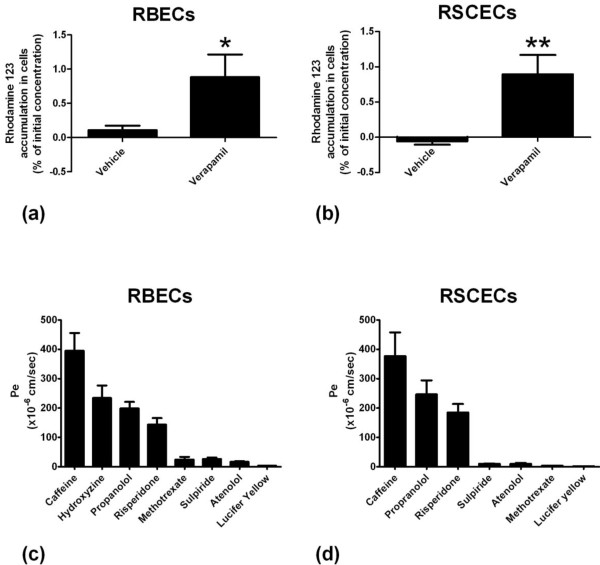
**Characterisation of optimised RBEC and RSCEC barriers for use in small molecules drug studies.** Intracellular accumulation of the fluorescent P-gp efflux transporter substrate rhodamine 123 in (**a**) RBECs (n = 4) and (**b**) RSCECs (n = 3) cultured on cell culture inserts in the absence and presence of the inhibitor verapamil. Data is presented as mean ± SEM and was analysed using an unpaired, two-tailed students *t*-test, *P < 0.01, **P < 0.001. Permeability coefficients for CNS-permeable and impermeable small molecule drugs were calculated across (**c**) RBEC (n = 5 independent cell culture experiments) and (**d**) RSCEC monolayers cultured on cell culture inserts (n = 3 independent cell culture experiments).

We next investigated the utility of the passaged RBEC and RSCEC monolayers for small molecule drug discovery purposes. To this end we analysed whether the optimised endothelial cells from rat brain and spinal cord exhibited functional P-gp efflux transporter activity and formed barriers that were discriminating to passage of small molecule compounds known to be excluded from the CNS parenchyma *in vivo*. To investigate the functionality of P-gp efflux transporter in the RBEC and RSCEC *in vitro* models we analysed the intracellular accumulation of rhodamine 123 in cells cultured on cell culture inserts. Rhodamine 123 is a fluorescent P-gp substrate which is actively effluxed from cells that express this clinically important transporter. Active efflux of rhodamine 123 can be reduced by treatment with the P-gp inhibitor verapamil, resulting in the accumulation of fluorescence within the cell. The RBEC and RSCEC monolayers both demonstrated negligible accumulation of rhodamine 123 when treated with DMSO vehicle (Figure 8a, b). Both cell types showed basal uptake of less than 0.1% of the initial input rhodamine 123 concentration. Pre-treatment of the cultured RBECs and RSCECs with 100 μM verapamil resulted in a significant increase in rhodamine 123 accumulation in both cell types (Figure 8a, b). Cellular uptake of rhodamine 123 increased to 0.88 ± 0.33% and 0.89 ± 0.27% for RBECs and RSCECs respectively. These observations indicate that P-gp efflux transporter is functional in RBEC and RSCECs co-cultured with mixed glia in the EBM-2/EGM-2 media formulation.

We next investigated whether the *in vitro* RBEC and RSCEC barriers were effective at preventing the passive permeability of small molecules known to be excluded from the CNS by the BBB and BSCB *in vivo*. Tight *in vitro* barriers are critical for such experiments, reducing paracellular permeability of small molecules which would otherwise lead to misleading estimates of passive permeability. We chose a panel of lipophilic small molecules known to cross the BBB/BSCB by passive diffusion *in vivo* (propanolol, caffeine, hydroxyzine and risperidone) and a panel of molecules with poor passive permeability characteristics that do not easily cross into the CNS *in vivo* (atenolol, methotrexate and sulpiride). Both the RBECs and the RSCECs had excellent discriminatory characteristics and excluded the poorly permeable molecules whilst allowing passage of the more lipophilic, CNS penetrant compounds (Figure 8c, d, Table 4). For the RBEC monolayer, the CNS penetrant molecules showed permeability coefficient values ranging from 143 to 394 × 10^-6^ cm/sec, with the poorly permeable compounds showing values 3.37 to 26.4 × 10^-6^ cm/sec. The CNS-crossing molecules showed permeability values of 184 to 376 × 10^-6^ cm/sec across the RSCEC monolayers, whilst the poorly permeable drugs showed values as low as 1.73 to 10.3 × 10^-6^ cm/sec. Thus the endothelial barriers formed by both RBECs and BSCB in the optimized EBM-2/EGM-2 culture conditions show excellent discriminatory characteristics that accurately predicted the permeability of CNS-crossing and impermeable small molecule drugs *in vitro*.

**Table 4:** Permeability of CNS and non-CNS crossing small molecules across RBEC and RSCEC monolayers grown on Millipore cell culture inserts

		**RBECs**			**RSCECs**		
	**Passive transport into CNS?**	**Number of inserts**	**Mean Pe (×10**^**-6**^ **cm/sec)**	**SEM (×10**^**-6**^ **cm/sec)**	**Number of inserts**	**Mean Pe (×10**^**-6**^ **cm/sec)**	**SEM (×10**^**-6**^ **cm/sec)**
Atenolol	No	15	16.53	2.939	6	9.788	3.166
Caffeine	Yes	9	394.9	60.46	6	376.4	81.13
Hydroxyzine	Yes	8	234.3	42.92	nd	nd	nd
Lucifer yellow	No	15	3.37	0.328	6	1.734	0.0734
Methotrexate	No	15	24.26	9.164	6	3.458	0.3547
Propanolol	Yes	15	198.8	22.16	6	246.5	47.94
Risperidone	Yes	11	143.1	22.54	6	184.5	29.01
Sulpiride	No	15	26.41	5.003	6	10.29	1.157

## Discussion

The development and improvement of *in vitro* models of the blood-CNS barriers is an ongoing effort towards both understanding the biology of these important regulatory tissues, and being able to overcome the formidable obstacle that they present to the delivery of therapeutics for the treatment of debilitating neurological diseases. Human, rat and mouse BBB cell lines have been developed but, although cheap and convenient to use, these cells produce barriers with high intrinsic paracellular permeability making them poorly-suited for applications such as drug transport screening and characterisation [5,76-78]. Due to this fact, *in vitro* primary cell models of highly differentiated endothelial cells from the BBB and BSCB remain a critical tool for investigative and pharmaceutical biology, particularly in the species typically used in pre-clinical studies. Such *in vitro* primary models have been utilised for genomics and proteomics studies [30,79-81], analysing endothelial transporter function [15,16,65,82,83], studying brain metastasis of cancer cells [84], and applied to translational pharmaceutical studies, investigating small molecule drug transport [77,85,86] and toxicity [67,87].

Here, we describe a further development in techniques producing such *in vitro* barrier models from primary rat CNS tissue, providing the first description of the provision of both brain and spinal cord endothelial cells from the rat, a species of pre-clinical importance in pharmaceutical CNS drug development and a commonly used laboratory model organism. High yields of differentiated cells are cultured from the same donor animals, reducing cost, labour and number of animals required. Furthermore, the endothelial cells obtained by this method are able to form monolayers with excellent barrier characteristics *in vitro*, making them suitable for use in biological investigations and in drug transport and toxicity studies. Importantly, our methods detail the first procedure for the culture of robust and *in vivo*-like spinal cord endothelial cells from the rat, complementing existing descriptions from mouse [55], and we also provide the first description of a functional *in vitro* barrier phenotype for spinal cord endothelial cells from any species.

Our aim was to achieve high yields of RBECs and RSCECs to provide a large number of cells that could be used for biological studies and drug discovery. We therefore introduced steps into our protocol to facilitate enhanced recovery of endothelial cells. Firstly, to increase cell numbers, we omitted size-dependent filtration from our microvessel isolation protocol. Filtering the microvessels enriches for smaller capillaries, which are hypothesised to be more “BBB-like” [88], but decreases the overall yield of endothelial cells. By filtering through 40 μm or 70 μm cell strainers, we observed that many microvessels of a wide range of sizes, including small capillaries, were retained on the cell strainer. We thus plated out the whole microvessel pellet isolated by BSA density centrifugation and subjected it to puromycin purification [27,31,51,61,65]. In addition to maintaining purity, the puromycin also enforces a selective pressure on the endothelial cells; RBECs and RSCECs with high expression of P-gp, a characteristic of *in vivo* blood-CNS barriers, are able to survive and proliferate *in vitro*. Indeed our data indicates that both RBECs and RSCECs in culture express P-gp efflux transporters (Figures 5h and 7h) that retain functionality (Figure 8a, b). By culturing cells using this selective method, we obtained large numbers of primary endothelial cells, which were then passaged onto cell culture inserts or tissue culture plastic and retained a highly-differentiated barrier phenotype.

We next looked to improve reproducibility in obtaining tight barriers, as a lack of robustness is a common problem when culturing primary brain endothelial cells *in vitro*. Our observations from passaging brain endothelial cells led us to an approach of splitting cells on the basis of the surface areas of the receiving culture dishes, rather than by traditional dilution splitting on the basis of cell numbers. If RBECs or RSCECs were transferred to new dishes or cell culture inserts as a dilution passage (e.g. splitting 1:4–1:2) they frequently grew as islands of cells which stopped proliferating and did not form a continuous monolayer, rendering them unsuitable for barrier studies on cell culture inserts. If, however, the cells were transferred on the basis of the surface area of the dish they were passaged into, they quickly reached confluence and formed functional barriers (Figure 1). Thus, a key element of our protocol is the concept of the ~1:1 passage of endothelial cells. This transfer method facilitated excellent barrier phenotypes for both RBECS and RSCECs (Figures 3, 4 and 6), with no obvious endothelial de-differentiation as judged by immunocytochemistry which demonstrated well organised, mature tight junctions and expression of endocytic transport machinery (Figures 5 and 7). Furthermore, the technique reliably resulted in the provision of useable barriers in almost every insert. We observed very low losses of individual inserts where barriers did not form, as often happened with dilution passaging. Individual inserts where the barrier failed were usually found to be due to handling technique and mechanical damage to the monolayer.

A major finding of our study was that barriers with high TEER and low Pe to small molecules such as LY were reproducibly obtained when culturing RBECs and RSCECs in Lonza’s EBM-2 basal medium with the EGM-2 BulletKit minus VEGF. The Lonza BulletKit contains supplements, such as hydrocortisone and FGF, that are well validated to improve endothelial barrier function *in vitro*[22,61]. This EBM-2/EGM-2 media combination outperformed DMEM supplemented with another commercial supplement, MVGS. The optimised conditions also included the addition of 15% plasma-derived serum and the monolayers did not display the sensitivity to serum-derived factors that has been observed in some *in vitro* BBB cell culture models [52].

Historically, the BBB has been more highly studied than the BSCB both *in vivo* and *in vitro*. An emerging consensus is that the two are broadly similar with some subtle differences, for example in their permeability and their vulnerability to certain insults and diseases [4,89]. The BSCB has an almost identical physical structure to that of the BBB, with tight junction-containing endothelial cells surrounded by and interacting with astrocytes and pericytes [89]. The BSCB also appears to be more permeable than the BBB in certain sub-regions but is still a tight and highly regulated barrier that protects the spinal cord parenchyma. Studies *in vivo* have indicated that the BSCB is more permeable than the BBB to small tracers, cytokines and neurotrophins, with lumbar regions of the spinal cord in particular being more permeable [90-94]. Some cytokines and growth factors however, such as IL-1α and granulocyte-macrophage colony-stimulating factor (GMCSF), have been shown to have similar transport across the BSCB as the BBB *in vivo*[95,96]. Other evidence suggests that the BBB and BSCB have similarly low permeability to large plasma proteins such as IgGs and albumin [95,97]. Similarities between the BBB and BSCB have also been noted in the expression and functionality of ABC transporters, which are hypothesised to play key roles in disease and drug resistance. Isolated capillaries from mouse brain and spinal cord show similar expression and functionality of P-gp, MDR2 and BRCP [82,83]. ABC-transporters at the BBB and BSCB also share similar increased expression and functionality following exposure to dioxins, and in mouse models of amyotropic lateral sclerosis (ALS) *in vivo*[82,83]. Interestingly, our models show that RSCEC monolayers are generally slightly more permeable than with RBECs, in spite of the fact that both are cultured in the presence of glial cells derived from brain tissue. Although data is lacking attesting to differences between brain and spinal cord astrocytes in their ability to induce barrier phenotypes, our observations may indicate that some aspects of the permeability properties of RSCECs are cell-intrinsic.

The only previously published *in vitro* study comparing BBB and BSCB cells was carried out using endothelial cells derived from mouse [55]. In that study, culture conditions for both endothelial cell types were established and expression levels of proteins associated with barrier function were characterised, although no functional barrier data was presented. Ge and Pachter (2006) found that cultured endothelial cells from both type of CNS tissue were indistinguishable under the microscope and showed identical expression of the endothelial markers vWF and PECAM-1, as well as similar uptake of LDL [55]. Our extensive characterisation data for endothelial markers, tight junction proteins, endocytic machinery and the P-gp efflux transporter, suggests a similar situation to be true for brain and spinal cord endothelial cells from rat (Figures 5 and 7). Additionally, Ge and Pachter provided a highly useful comparison of several genes important for barrier function in these cultured endothelial cells [55]. Gene expression of claudin-1, claudin-5, P-gp and transferrin receptor were unchanged between both types of endothelial cell in mouse, but expression levels of ZO-1, occludin, β-catenin and VE-cadherin were lower in spinal cord endothelial cells compared to those from brain tissue [55]. This observation is in agreement with *in vivo* descriptions of the BSCB being more permeable than the BBB.

Our observations further support and extend these observations on the structure and function of the BBB and the BSCB. We have shown that cells from both the rat BBB and BSCB can be cultured on cell culture inserts *in vitro* to form functionally restrictive cell monolayers, with the endothelial cells of the brain forming slightly tighter barriers than those of the spinal cord. In this regard our *in vitro* models apparently mimic the *in vivo* situation for the BBB and BSCB. We observed an excellent relationship between pre-experimental TEER values and Pe to LY in permeability assays for both models (R^2^ = 0.78 for RBECs and 0.91 for RSCECs). Importantly, this indicates that the pre-experimental TEER value is predictive of the Pe to LY, allowing consistent and reproducible experiments to be performed. Cell culture inserts with high TEER values can be selected from the outset and matched with inserts of similar barrier tightness, allowing robust experiments to be performed on primary-derived cells that have similar intrinsic permeability properties.

Our BBB and BSCB models exhibited excellent discrimination characteristics for limiting the passage of small and large molecules that cross the barrier by paracellular diffusion, such as LY and FITC-dextrans, and also for small molecules that enter the CNS poorly on the basis of their low lipophilicity. This makes our models ideally suited for *in vitro* permeability studies, particularly for small molecule drugs where tight *in vitro* barriers are critically required to minimise non-specific paracellular transport that would mask true permeability characteristics and kinetics. Small molecule permeability across an *in vitro* barrier has been demonstrated by several groups using different species, including human, porcine, mouse and rat, but only across endothelial monolayers derived from cells of the BBB [16,25,54,65]. Our methods show that we are able generate a large number of tight *in vitro* barriers representing the rat BBB, but we also demonstrate for the first time an *in vitro* model of rat spinal cord endothelial cells that shows similar restrictive properties to small molecules. These data suggest that our models would be suitable for a broad range of CNS drug discovery studies, particularly for instances where a drug target is located within the spinal cord as well as, or instead of, in the brain. The RBEC and RSCEC barriers also show expression and functionality of the clinically important efflux transport P-gp (Figures 5h, 7h and 8a, b). These BBB and BSCB models could thus be used for *in vitro* studies of barrier function involving this transporter, such as determining the efflux of chemotherapeutic small molecule drugs which are often also P-gp substrates.

Since differences between the BBB and BSCB exist, it is therefore essential that *in vitro* models for both barriers are available for research purposes. An *in vitro* model for one barrier may not necessarily be an appropriate substitute for the other. This may be of particular relevance when studying diseases which affect one CNS compartment more than the other [4,89]. Our *in vitro* models of both types of blood-CNS barrier are thus of great potential value for the investigation of such disease processes. Since these models are optimised for rat tissues, a species for which relevant and well-characterised *in vivo* models of CNS-disease exist, they have great potential utility in translational studies. Our novel *in vitro* RSCEC model of the rat BSCB may also contribute to the furthering of knowledge about this poorly-understood blood-CNS barrier and could be applied to genomics and proteomics studies in the future.

## Conclusion

In conclusion, we have demonstrated an easy and robust method to prepare large yields of endothelial cells from rat brain and spinal cord tissue. Our method has the advantages of ease-of-use and reproducibility and provides culture conditions suitable for the isolation and culture of both RBECs and RSCECs as coincident sister cultures. The high yields of cells obtained go some way to overcoming the often limiting amount of material available for experiments, a common problem that is often encountered when performing studies with primary cell models of the BBB and BSCB. The optimised RBEC and RSCEC cultures show expression of typical markers representative of the blood-CNS barriers *in vivo* and form functional barriers *in vitro* that are discriminating in preventing the passage of large molecules and poorly lipophilic small molecule drugs. The tight barrier phenotype obtained for both models allows predictive drug permeability studies to be performed, due to the low intrinsic non-specific paracellular permeability of the pure endothelial monolayers. We hope that these models will prove to be a valuable addition to the tools available to academic and industrial researchers for both drug discovery and studying the biology of BBB and BSCB in an *in vitro* setting.

## Abbreviations

BBB: Blood–brain barrier; BSCB: Blood-spinal cord barrier; RBEC: Rat brain endothelial cells; DIV: Days *in vitro*; RSCEC: Rat spinal cord endothelial cells; TEER: Transendothelial electrical resistance; MVGS: Microvascular growth supplement; LY: Lucifer yellow; DLS: Dynamic light scattering; Pe: Permeability coefficient; vWF: Von Willebrand factor; SMA: Smooth muscle actin; PDGF: Platelet-derived growth factor; PDS: Plasma-derived bovine serum; PECAM-1: Platelet/endothelial cell adhesion molecule-1; LDL: Low density lipoprotein; TLCK: Tosyl-lysine-chloromethylketone; LC-MS/MS: Liquid chromatography mass spectrometry; P-gp: P-glycoprotein; FGF: Fibroblast growth factor; FITC: Fluorescein isothiocyanate; CNS: Central nervous system.

## Competing interests

PMDW, JP, GT and CW are employees of MedImmune Ltd, an AstraZeneca PLC owned company. At the time of the study SL and UG were employees of AstraZeneca PLC.

## Authors’ contributions

PMDW and JP designed and performed all experimental work (with the exception of LC-MS/MS) and wrote the manuscript. SL and UG assisted with the design of the small molecule permeability study and UG performed LC/MS experiments. PMDW, JP, GT, SL, UG and CIW analysed and interpreted experimental data. All authors have approved the final version of the manuscript.
